# Interpreting the Global Enteric Multicenter Study (GEMS) Findings on Sanitation, Hygiene, and Diarrhea

**DOI:** 10.1371/journal.pmed.1002011

**Published:** 2016-05-03

**Authors:** Jonny Crocker, Jamie Bartram

**Affiliations:** 1 The Water Institute, University of North Carolina at Chapel Hill, Chapel Hill, North Carolina, United States of America; 2 Department of Environmental Sciences and Engineering, University of North Carolina at Chapel Hill, Chapel Hill, North Carolina, United States of America

## Abstract

In this Perspective on the GEMS study by Kelly Baker and colleagues, Jonny Crocker and Jamie Bartram consider the implications of associations found and not found between diarrheal disease and sanitation and hygiene.

Sanitation and hygiene are global concerns, as reflected in international development and human rights policy [1,2]. The Sustainable Development Goals (SDGs) include target 6.2: to “achieve access to adequate and equitable sanitation and hygiene for all and end open defecation” [3]. Globally, about 2.5 billion people do not use improved sanitation, of whom 1 billion defecate in the open [4]. Fecal contamination of the environment and poor handwashing are responsible for an estimated 577,000 deaths annually [5]. This is likely an underestimation: there is emerging evidence that poor sanitation and hygiene contribute to undernutrition [6,7] and could be responsible for approximately half of all child stunting [8–10]. Much of the health impact of inadequate sanitation and hygiene is attributed to diarrheal disease and its secondary effects. However, diarrhea is difficult to measure, and sanitation and hygiene are difficult to link to health outcomes [11].

## The Global Enteric Multicenter Study

In this issue of *PLOS Medicine*, Kelly Baker and colleagues report on the associations between sanitation and hygiene indicators and moderate-to-severe diarrhea (MSD) [12]. Their Global Enteric Multicenter Study (GEMS) collected data on MSD among children reporting to health centers in seven sites in seven countries from 2007 to 2011, with cases matched to controls by village and homes visited within 90 days to observe sanitation and hygiene conditions. The authors report that, at four of the seven sites, access to shared sanitation compared to private sanitation was a risk factor for MSD. At one site (in Bangladesh), shared sanitation was associated with lower risk of diarrhea. Lack of access to sanitation was a risk factor for MSD only at the Kenya site, where 29.7% of cases lacked sanitation access. The remaining six sites had 0%–7.6% of cases from households without sanitation access, which limited the power to detect an associated risk at these sites. Interestingly, other indicators such as child feces disposal in the open and visible feces in the area were not associated with MSD at more than a single site.

## Study Design and Sanitation and Hygiene Indicators

Diarrheal data for GEMS were clinically and laboratory confirmed, making it one of the highest-quality health outcome datasets associated with any sanitation or hygiene study. However, there are a number of limitations to the sanitation and hygiene indicators that suggest caution in interpreting the findings.

GEMS sanitation and hygiene indicators are at the household, not individual, level and are indicators of access, not behavior (except child feces disposal). Access does not equate to use, and behaviors within a household often vary, for example, by age and gender [13]. Survey best practice is to inquire about individual behaviors both at and away from home, in addition to observing sanitation facilities [14]. Likewise, the link between available handwashing materials and behaviors is not a given [15].

GEMS did not assess neighborhood- or village-level sanitation, so they could not be controlled for. This also prevented assessment of externalities, which may occur with shared sanitation. A study from India reported that infant mortality externalities from neighborhood sanitation were larger than benefits from household-level access [16].

GEMS is nonexperimental, so omitted variable bias is possible. The authors note that shared sanitation is more common in densely populated areas. These are also plausibly where households lack yards, so children play outside around drainage ditches, experiencing greater risk of exposure to fecal matter.

Observation of sanitation and hygiene conditions occurred up to 90 days after children presented with MSD. While access indicators are unlikely to change over such a short interval, available handwashing materials and feces in the area could change in shorter periods. Direct observation of feces, as was undertaken, could logically associate with MSD; Baker and colleagues’ failure to detect a significant association should be interpreted cautiously, given the exposure variable was observed well after MSD cases.

Sanitation and hygiene health outcomes are not limited to diarrhea. Asymptomatic enteric infections are common among children in developing countries [17] and may contribute to malnutrition and stunting [18]. While GEMS did not assess these outcomes, where shared sanitation is a risk factor for diarrhea, it is also likely a risk factor for other health outcomes.

## Variability and Context

The link between sanitation, hygiene, and health is mediated by local behaviors and environmental conditions. Baker and colleagues report site-by-site results, contributing to understanding variability and informing recommendations for different settings, which is more valuable than generalization. However, there is little discussion of the variation in results. Site descriptions would have been valuable. Did sites where shared sanitation was a risk factor have notable characteristics in common? Analysis of sanitation and hygiene interactions would also have been interesting, though limited by the near-uniform indicators at some sites. Was shared sanitation a greater risk factor where handwashing materials were lacking or where feces were visible at the defecation site?

## Concluding Remarks

The draft sanitation ladder for measuring SDG progress allows sharing of improved facilities by fewer than five households to count towards ending open defecation [19]. Higher rungs refer to private facilities and safe excreta management. The indicators also interpret access as including use, which was not included in GEMS. Future research should include indicators on use of facilities and excreta management.

Baker and colleagues provide valuable evidence that confirms that private sanitation often provides greater benefits than shared sanitation. Prior evidence suggests health benefits for use of any sanitation facility (including shared) when compared to open defecation [8–10]. This study will inform policy and programming, yet shared facilities may still have a role in addressing open defecation in challenging settings. For reasons beyond just health such as dignity and gender equity [20,21], we should advocate for private access whenever possible.

Baker and colleagues present the best dataset yet on diarrheal disease associated with sanitation and hygiene. They provide compelling evidence on sanitation and hygiene risk factors for MSD and variability in that risk. Importantly, they also demonstrate the feasibility and value of rigorous data collection on health outcomes, something that future studies should develop yet further.

## Abbreviations

GEMS: Global Enteric Multicenter Study
MSD: moderate-to-severe diarrhea
SDG: Sustainable Development Goal
